# Prognostic significance of the controlling nutritional status (CONUT) score in patients undergoing hepatectomy for hepatocellular carcinoma: a systematic review and meta-analysis

**DOI:** 10.1186/s12876-019-1126-6

**Published:** 2019-12-09

**Authors:** Kosei Takagi, Piotr Domagala, Wojciech G. Polak, Stefan Buettner, Jan N. M. Ijzermans

**Affiliations:** 1 Department of Surgery, Erasmus MC, University Medical Center Rotterdam, Dr. Molewaterplein 40, 3015 GD Rotterdam, The Netherlands; 2 Department of Gastroenterological Surgery, Okayama University Graduate School of Medicine, Dentistry, and Pharmaceutical Sciences, Okayama, Japan; 3 Department of General and Transplantation Surgery, The Medical University of Warsaw, Warsaw, Poland

**Keywords:** Controlling nutritional status (CONUT) score, Hepatocellular carcinoma, Outcome, Meta-analysis

## Abstract

**Background:**

The clinical value of the controlling nutritional status (CONUT) score in hepatocellular carcinoma (HCC) has increased. The aim of this meta-analysis was to systematically review the association between the CONUT score and outcomes in patients undergoing hepatectomy for HCC.

**Methods:**

Embase, Medline Ovid, Web of Science, Cochrane CENTRAL, and Google Scholar were systematically searched. Random effects meta-analyses were conducted to examine the prognostic value of the CONUT score in HCC patients.

**Results:**

A total of five studies including 4679 patients were found to be eligible and analyzed in the meta-analysis. The CONUT score was significantly associated with overall survival (HR 1.78, 95%CI = 1.20–2.64, *P* = 0.004, *I*^*2*^ = 79%), recurrence-free survival (HR 1.34, 95%CI = 1.17–1.53, *P* < 0.001, *I*^*2*^ = 16%) and postoperative major complications (OR 1.85, 95%CI: 1.19–2.87, *P* = 0.006, *I*^*2*^ = 72%) in HCC patients. Moreover, the CONUT score was associated with the Child–Pugh classification, liver cirrhosis, ICGR15, and tumor differentiation. However, it was not associated with tumor size, tumor number, and microvascular invasion.

**Conclusions:**

The CONUT score is an independent prognostic indicator of the prognosis and is associated with postoperative major complications and hepatic functional reserve in HCC patients.

## Background

Hepatocellular carcinoma is a major cause of cancer-related morbidity and mortality [1]. Despite advances in early diagnosis and personalized medicine, the clinical outcome of hepatocellular carcinoma (HCC) remains poor with high recurrence rate after curative treatment [2]. Therefore, the identification of accurate and reliable prognostic markers is necessary in HCC patients.

The controlling nutritional status (CONUT) score [3], calculated from serum albumin level, total cholesterol level, and total lymphocyte count, was originally developed as a nutritional assessment tool in Western Europe in 2005. The evidence regarding the influence of the CONUT score on prognosis in gastrointestinal cancers has been growing, particularly in Asian populations [4, 5]. We have recently reported on the association between the CONUT score and postoperative complication risk in gastrointestinal and hepato-pancreato-biliary surgical oncology [6]. However, to the best of our knowledge, no study has systematically investigated the significance of the CONUT score on outcomes in patients with HCC. Surgical complication risk and cancer prognosis differ between cancers and procedures. Therefore the effect of the CONUT score in patients with specific cancers should be systematically examined separately.

We herein conducted this systematic review and meta-analysis to evaluate the association between preoperative CONUT score and outcomes in patients undergoing hepatectomy for HCC. Furthermore, the impact of the CONUT score on clinicopathological factors was identified score in HCC patients.

## Methods

### Search methodology

A systematic literature search was performed on July 4th 2019 in 5 publication repositories: Embase, Medline Ovid, Web of Science, Cochrane CENTRAL, and Google scholar. The full search for all repositories is appended to this article (Additional file 1: Table S1). The methods for developing our search have been detailed in a previous publication [6]. This study is reported according to the Preferred Reporting Items for Systematic Reviewers and Meta-Analyses (PRISMA) guidelines [7].

### Criteria for the review

Inclusion criteria were the following: original article, patients undergoing hepatectomy for HCC; the preoperative assessment of the CONUT score; and reported postoperative outcomes. In the case of multiple publications by the same institute, the study focusing on long-term outcomes or the study with the last date of publication was included in the meta-analyses.

Titles, abstracts and full articles were screened independently by two investigators (KT and PD), according to the PRISMA guidelines. All original articles that met the criteria were included. From the included articles, year and country of study publication, study type, patient information, cut-off and prevalence of the CONUT score, and postoperative short-term and long-term outcomes were extracted. The methodological quality of each studies was evaluated based on the Newcastle-Ottawa quality assessment scale for cohort studies [8]. Studies with a total score with 6 or higher were considered high-quality studies [9].

The primary outcomes were overall survival (OS), defined as time from surgery to death or last follow-up, and recurrence-free survival (RFS), defined as time from surgery to recurrence or last follow-up/death. Secondary outcomes were postoperative complications and the clinicopathological parameters. Postoperative complications were graded based on the Clavien–Dindo classification (CDc) [10], with major complications defined as CDc ≥3. The clinicopathological parameters included the Child–Pugh classification (A versus B), the degree of liver cirrhosis, indocyanine green retention test after 15 min (ICGR15), and tumor characteristics (tumor size, tumor number [single versus multiple], tumor differentiation [well and moderate differentiated versus poorly differentiated], and microvascular invasion).

### Statistical analysis

Random effects meta-analyses were conducted to estimate the average correlation of the CONUT score with OS and RFS. Random effects models were used, as the populations were heterogeneous and consisted of patients deriving from different countries and undergoing different treatment protocols. The pooled hazard ratio (HR) with 95% confidence interval (95%CI) and the mean difference (MD) for continuous variables with 95%CI were calculated using the inverse variance method. The pooled odds ratio (OR) for dichotomous variables was calculated using the Mantel-Haenszel method. Heterogeneity among studies was quantified by calculating the *I*^*2*^ values and the Chi-square test, with *P* < 0.05 being statistically significant and *I*^*2*^ values of 50% or more indicating the presence of heterogeneity. Potential publication bias for outcomes was examined using Funnel plots. Analyses were conducted using R 3.5.4 (cran.r-project.org) and Review Manager 5.3 (Cochrane Collaboration, 2014).

## Results

### Characteristics of included studies

**Fig. 1:**
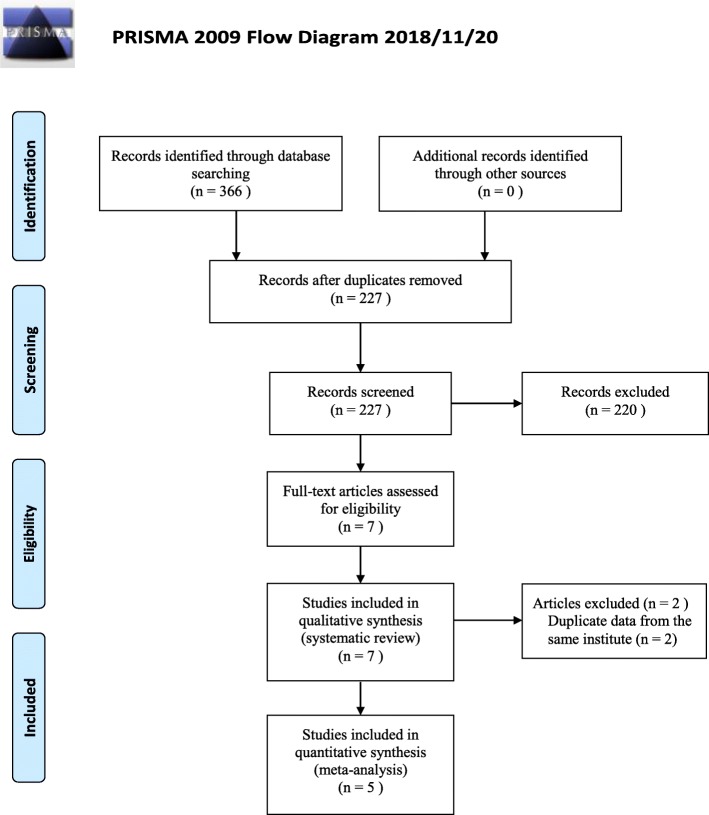
PRISMA 2009 Flow Diagram

From 366 retrieved clinical records, seven articles met the inclusion criteria (Fig. 1) [11–17]. All included studies were retrospective series from Asian countries, more specifically Japan and China (Table 1). There were six single center studies [11–13, 15–17] and one multi-center study [14]. Regarding the cut-off value of the CONUT score, six studies used preoperative CONUT score with the following cut-off values: CONUT ≤1 vs CONUT ≥2 in one study [17], CONUT ≤2 vs CONUT ≥3 in two studies [11, 16], and CONUT ≤3 vs CONUT ≥4 in two studies [12, 14], and CONUT ≤4 vs CONUT ≥5 in one study [13]. While postoperative CONUT score was used in one study with the cut-off value of CONUT ≤7 vs CONUT ≥8 [15]. The prevalence in patients with high CONUT score ranged between 19 and 49%. Five studies [11, 12, 14, 16, 17] focused on long-term outcome as primary endpoints, and two studies [13, 16] focused on short-term outcome. The quality assessment of the included studies found that all studies were considered to be of high-quality with a score of 6 or higher based on the Newcastle-Ottawa quality scale (details shown in Additional file 1: Table S2).

**Table 1:** Literatures of the effects of the CONUT score in patients undergoing hepatectomy for hepatocellular carcinoma

Study	Year	Country	Study design	Number (Male)	Tumor stage	Cut-off for high CONUT group	Prevalence of high CONUT score	End points	Quality^a^
Takagi et al. [11]	2017	Japan	Retrospective Single center	295 (241)	I: 36 II: 126 III: 92 IV: 41	≥3	40.0%	OS RFS	7
Harimoto et al. [12]	2017	Japan	Retrospective Single center	357 (270)	I: 58 II: 187 III: 93 IV: 19	≥4	19.3%	OS RFS	6
Takagi et al. [13]	2018	Japan	Retrospective Single center	331 (269)	I + II: 185 III + IV: 146	≥5	9.1%	Complications	6
Harimoto et al. [14]	2018	Japan	Retrospective Multi-center	2461 (1785)	I + II: 1437 III + IV: 1024	≥4	21.9%	OS RFS	6
Li et al. [15]	2018	China	Retrospective Single center	1334 (1136)	n.a.	≥8^b^	49.4%	Complications	8
Wang et al. [16]	2018	China	Retrospective Single center	209 (172)	BCLC stage A: 126 B: 40 C: 43	≥3	34.5%	OS RFS PHR	6
Lin et al. [17]	2019	China	Retrospective Single center	380 (333)	I + II: 304 III + IV: 76	≥2	49.2%	OS RFS	8

^a^Score from a maximum of 9 evaluated by the Newcastle–Ottawa quality assessment scale for cohort studies [8].

^b^Evaluated by postoperative CONUT score

*CONUT* controlling nutritional status, *OS* overall survival, *RFS* recurrence-free survival, *n.a* not available, *BCLC stage* Barcelona clinic liver cancer stage, *PHR* postoperative hepatitis B virus reactivation

### Reported outcomes

The literatures reporting the effects of the CONUT score on outcome in patients undergoing hepatectomy for HCC are summarized in Table 2.

**Table 2:** Literatures reporting the effects of the CONUT score on postoperative outcome in patients undergoing hepatectomy for hepatocellular carcinoma

Study	Complications	Mortality	Recurrence-free survival	Overall survival
Takagi et al. [11]	Major (CDc ≥ III): 15 vs 14% (*P* = 0.79)	n.a.	5-year: 27.9 vs 41.4% (*P* = 0.011) HR 1.64 (1.15–2.30), *P* = 0.006^a^	5-year: 61.9 vs 74.9% (*P* = 0.006) HR 2.50 (1.47–4.23), *P* = 0.001^a^
Harimoto et al. [12]	Major (CDc ≥ III): 20.3 vs 14.9% (*P* = 0.36)	n.a.	5-year: 8.8 vs 38.0% (*P* < 0.01) HR 1.51 (1.06–2.15), *P* = 0.02^b^	5-year: 47.6 vs 78.0% (*P* < 0.01) HR 2.16 (1.25–3.72), *P* = 0.03^a^
Takagi et al. [13]	Overall (CDc ≥ II): 56.7 vs 45.5% (*P* = 0.24) Major (CDc ≥ III): 23.3 vs 13.6% (*P* = 0.15)	10.0 vs 1.3% (*P* = 0.002) OR 9.41 (1.15–77.4), *P* = 0.038^a^	n.a.	n.a.
Harimoto et al. [14]	Major (CDc ≥ III): 17.7 vs 11.0% (*P* < 0.01)	n.a.	HR 1.219 (1.06–1.40), *P* = 0.006^a^	HR 1.223 (1.06–1.41), *P* = 0.006^a^
Li et al. [15]	Major (CDc ≥ III): 15.6 vs 6.2% (*P* < 0.001) OR 2.05 (1.37–3.01), *P* < 0.001^a^	2.6 vs 0.4% (*P* = 0.001)	n.a.	n.a.
Wang et al. [16]	Overall: 74.3 vs 59.3% (*P* = 0.029)	n.a.	5-year: 10.0 vs 9.6% (*P* = 0.001) HR 1.54 (1.10–2.16), *P* = 0.011^a^	5-year: 31.3 vs 44.0% (*P* < 0.001) HR 1.62 (1.05–2.51), *P* = 0.03^a^
Lin et al. [17]	Overall (CDc ≥ II): 29.4 vs 23.3% (*P* = 0.177)	n.a.	5-year: 37.2 vs 47.6% (*P* = 0.016) HR 1.36 (1.00–1.85), *P* = 0.052^a^	5-year: 66.7 vs 82.8% (*P* < 0.001) HR 2.40 (1.74–4.25), *P* = 0.001^a^

Data are shown for high CONUT group versus low CONUT group. Odds ratio (OR) and Hazard ration (HR) is shown with 95% confidence interval. ^a^Multivariable analysis. ^b^Univariate analysis

*CONUT* controlling nutritional status, *CDc* Clavien–Dindo classification, *n.a* not available

Five studies reported data on OS and RFS [11, 12, 14, 16, 17]. The Kaplan-Meier curve showed that patients with preoperative high CONUT score had a significantly poorer prognosis in terms of OS and RFS than those with low CONUT score in all studies. In the multivariable analyses, preoperative CONUT score was identified as an independent predictor associated with OS in all five studies. Regarding RFS, three studies [11, 14, 16] showed a significant association between preoperative CONUT score and RFS in the multivariable analysis, whereas two studies [12, 17] showed no significant association.

All studies reported data on postoperative complications including major complications in five studies [11–14, 16], overall complications in three studies [13, 16, 17] and mortality in two studies [13, 15]. Takagi et al. [11, 13] showed no significant association between preoperative CONUT score and overall and major complications, but found the preoperative CONUT score to be associated with an increased risk of mortality after hepatectomy (OR 9.41, 95%CI = 1.15–77.4, *P* = 0.038). In addition, a higher CONUT score was related to the incidence of postoperative ascites, posthepatectomy liver failure, sepsis and enteritis. Harimoto et al. [14] reported significant differences between the groups in terms of major complications based on multi-center analysis (low CONUT: 11.0% vs. high CONUT: 17.7%; *P* < 0.01). Li et al. [15] demonstrated that early postoperative CONUT score was independently associated with major complications (OR 2.05, 95% CI = 1.37–3.01, *P* < 0.001). The CONUT score was also associated with postoperative pulmonary complications, bile leakage, intra-abdominal hemorrhage and posthepatectomy liver failure (grade C). Wang et al. [16] showed significant differences between the groups in overall complications (low: 59.3% vs. high: 74.3%; *P* = 0.029), however Lin et al. [17] found no significant differences in overall complications (low: 23.3% vs. high: 29.4%; *P* = 0.177).

Data on hepatic functional reserve and pathological findings including the degree of hepatic cirrhosis were examined in three studies [11, 12, 14]. Takagi (2017) et al. [11] reported that the CONUT score was significantly associated with platelet count (normal nutrition: 19.7 × 10^4^/μL vs. light undernutrition: 16.9 × 10^4^/μL vs. moderate undernutrition: 18.0 × 10^4^/μL; *P* = 0.003), prothrombin time (normal: 104% vs. light: 96% vs. moderate: 90%; *P* < 0.001), Child–Pugh classification grade B (normal: 2.4% vs. light: 0.6% vs. moderate: 5.4%; *P* = 0.01), technetium-99 m-galactosyl human serum albumin, and the hepatic cirrhosis (normal: 39% vs. light: 51% vs. moderate: 66%; *P* = 0.017). Harimoto (2017) et al. [12] found the significant association between the CONUT score and prothrombin time (low CONUT: 90% vs. high CONUT: 83%; *P* < 0.01), the Child–Pugh classification grade B (low: 4% vs. high: 26%; *P* < 0.01), and liver damage grade B (low: 13% vs. high: 50%; *P* < 0.01), but no association with ICGR15 (low: 14% vs. high: 16%; *P* = 0.12), hepatic cirrhosis (low: 41% vs. high: 52%; *P* = 0.11), and tumor characteristics. Harimoto (2018) et al. [14] demonstrated significant differences regarding total bilirubin (low: 0.79 mg/dl vs. high: 0.84 mg/dl; *P* < 0.01), prothrombin time (low: 93% vs. high: 85%; *P* < 0.01), ICGR15 (low: 15% vs. high: 20%; *P* < 0.01), the Child–Pugh classification grade B (low: 2% vs. high: 22%; *P* < 0.01), and hepatic cirrhosis (low: 47% vs. high: 64%; *P* < 0.01), however no differences regarding tumor characteristics. Li et al. [15] showed no differences of Child–Pugh classification grade B (low: 2.5% vs. high: 3.3%; *P* = 0.37), tumor characteristics, but hepatic cirrhosis (low: 54% vs. high: 64%; *P* < 0.001). Wang et al. [16] investigated the effect of the CONUT score in predicting postoperative hepatitis B reactivation (PHR). They found that the incidence of PHR was significantly higher in patients with high CONUT score (low: 5% vs. high: 32%; *P* < 0.001) and the CONUT score was strongly associated with PHR (HR 7.66, 95%CI: 2.47–23.8, *P* < 0.001) in the logistic regression model. Lin et al. [17] constructed the nomogram including the CONUT score, liver cirrhosis, tumor size and differentiation as prognostic variables. They reported the CONUT-based nomogram (the C-index 0.71, 95%CI: 0.65–0.77) had superior discriminative ability to predict overall survival compared with conventional staging systems such as the BCLC stage (the C-index 0.63, 95% CI: 0.58–0.68), the TNM classification (the C-index 0.59, 95% CI: 0.53–0.64), and the CLIP score (the C-index 0.58, 95% CI: 0.53–0.64).

### Meta-analysis

From seven studies included in the systematic review, two articles were excluded in the meta-analysis as duplicate data from the same institute was reported. Accordingly, five articles with 4679 patients were included in the meta-analysis [11, 14–17].

**Fig. 2:**
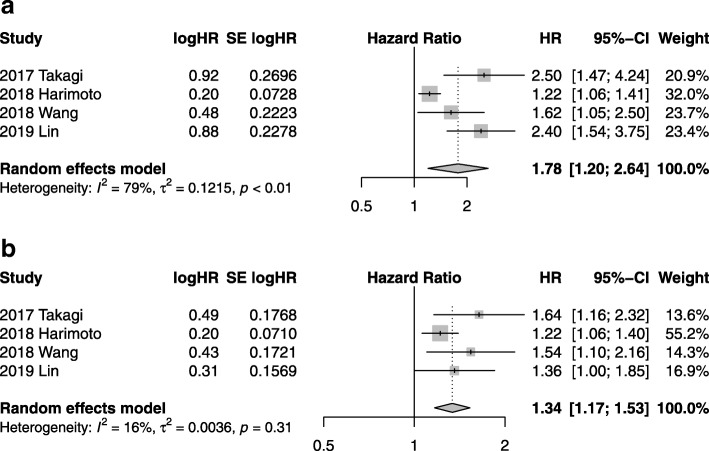
Forest plots demonstrating primary outcomes in terms of high CONUT group versus low CONUT group. **a** Overall survival; and (**b**) Recurrence-free survival

Based on four studies including 3345 patients [11, 14, 16, 17], patients with high CONUT score had a significantly worse OS and RFS compared with those with low CONUT score (OS: HR 1.78, 95%CI = 1.20–2.64, *P* = 0.004, *I*^*2*^ = 79%, *P* < 0.01; RFS: HR 1.34, 95%CI = 1.17–1.53, *P* < 0.001, *I*^*2*^ = 16%, *P* = 0.31), as shown in Fig. 2. A significant heterogeneity was found across the studies with respect to OS. Funnel plots of OS and RFS are demonstrated in Additional file 1: Figure S1.

**Fig. 3:**
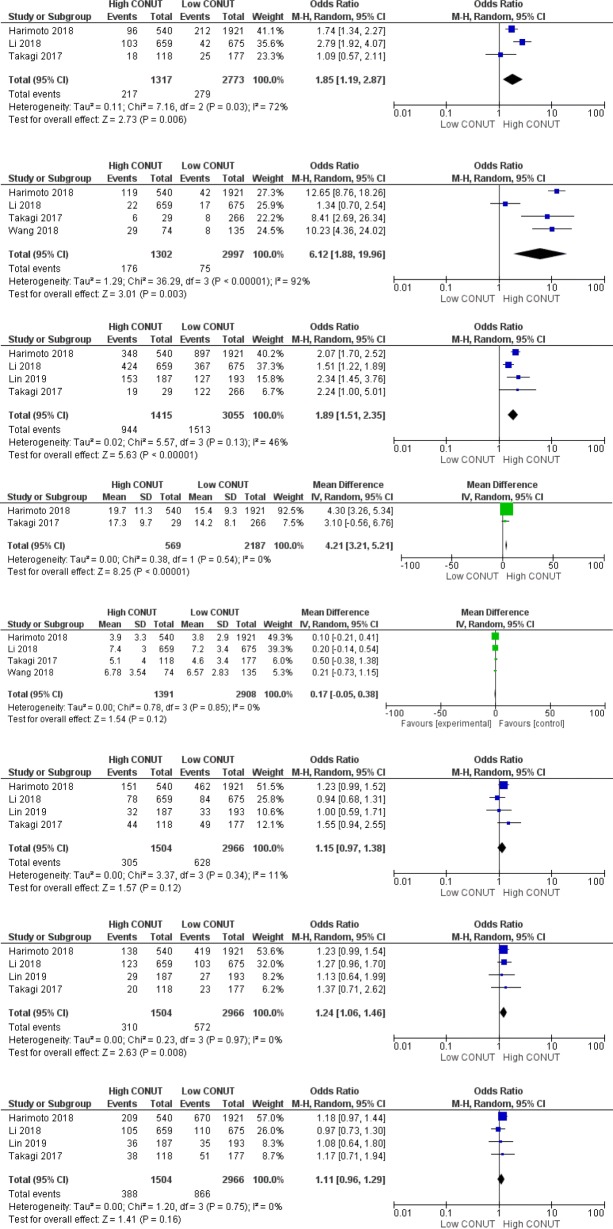
Forest plots demonstrating secondary outcomes in terms of high CONUT group versus low CONUT group. **a** Major complications (CDc ≥ III); (**b**) Child-Pugh B; (**c**) Liver cirrhosis; (**d**) ICGR15; (**e**) Tumor size; (**f**) Tumor number (multiple); (**g**) Poor differentiation; and (h) Microvascular invasion

Figure 3 shows results of the meta-analyses for the secondary outcomes in terms of high CONUT group versus low CONUT group. Meta-analyses showed that the CONUT score was associated with the incidence of postoperative major complications (OR 1.85, 95% CI: 1.19–2.87, *P* = 0.006, *I*^*2*^ = 72%, *P* = 0.03), the Child–Pugh classification B (OR 6.12, 95% CI: 1.88–20.0, *P* = 0.003, *I*^*2*^ = 92%, *P* < 0.001, liver cirrhosis (OR 1.89, 95% CI: 1.51–2.35, *P* < 0.001, *I*^*2*^ = 46%, *P* = 0.13), and ICGR15 (MD 4.21, 95%CI: 3.21–5.21, *P* < 0.001, *I*^*2*^ = 0%, *P* = 0.54). Regarding tumor characteristics, the CONUT score was associated with tumor differentiation (OR 1.24, 95% CI: 1.06–1.46, *P* = 0.008, *I*^*2*^ = 0%, *P* = 0.97). However, no significant association was found in tumor size (MD 0.17, 95% CI: − 0.05–0.38, *P* = 0.12, *I*^*2*^ = 0%, *P* = 0.85), tumor number (OR 1.15, 95% CI: 0.97–1.38, *P* = 0.12, *I*^*2*^ = 11%, *P* = 0.34), and microvascular invasion (OR 1.11, 95% CI: 0.96–1.29, *P* = 0.16, *I*^*2*^ = 0%, *P* = 0.75).

## Discussion

This systematic review and meta-analysis investigated the prognostic value of the CONUT score in patients undergoing hepatectomy for HCC. The present study demonstrated that the CONUT score was associated with OS, RFS and the incidence of postoperative major complications in patients with HCC. Moreover, we found that the CONUT score was associated with the Child–Pugh classification, liver cirrhosis, ICGR15, and tumor differentiation, whereas it was not associated with tumor size, tumor number, and microvascular invasion.

Recent meta-analyses have shown that the nutritional status evaluated by the CONUT score and the prognostic nutritional index (PNI) was associated with prognosis of various cancers [4, 5, 18]. Regarding the prognostic value of such nutritional assessment tools in HCC patients, recent meta-analyses have reported the relationship between the PNI and prognosis [9, 19]. However, the CONUT score has been reported to provide the most appropriate sensitivity and specificity in patients with HCC compared with other immune-nutritional parameters including the PNI [15, 17]. To date, the effect of the CONUT score on prognosis in patients with HCC has not been examined systematically. Actually, previous studies on the CONUT score in HCC patients reported different outcomes in terms of RFS and postoperative complications, as is shown in Table 2. Therefore, our results would add the clinical evidence of the association between the CONUT score and outcome in patients with HCC.

The present meta-analysis indicates that the CONUT score is associated with the prognosis, the postoperative major complications and hepatic functional reserve in HCC patients. Patients with high CONUT score had a significantly worse OS and RFS, and had a higher incidence of postoperative major complications than those with low CONUT score in HCC patients after hepatectomy. These results are in line with discovered correlations between nutritional status markers like PNI and sarcopenia, and the prognosis and postoperative complications in gastrointestinal and hepatopancreatobiliary surgical oncology [18, 20, 21]. In addition, it should be noted that prognosis in patients with HCC depends on tumor stage as well as hepatic functional reserve [22, 23]. Indeed, our meta-analysis demonstrated the relationship between the CONUT score and the Child–Pugh classification, liver cirrhosis, and ICGR15. Interestingly, Wang et al. reported that the CONUT score is an effective indicator predicting PHR in hepatitis B HCC patients [16]. Among tumor characteristics, tumor differentiation was the only pathological feature associated with the CONUT score.

The biological mechanism explaining the correlation between the CONUT score and short- and long-term outcomes is unknown. In past studies, preoperative higher CONUT score was found to be associated with worse nutritional status as well as poorer immune functional status preoperatively [16]. In addition, postoperative immune functional status was worse in patients with preoperative higher CONUT score. Perioperative poor immune-nutritional status could in turn be related to a higher incidence of postoperative complications. Separate CONUT score parameters have been correlated with outcomes in HCC patients in past studies. Serum albumin, on itself a major indicator of nutritional status, is associated with prognosis and complication risk in patients following hepatectomy for HCC [24, 25]. Total lymphocyte count is a surrogate marker of immune-nutritional status in cellular and antiviral immunity and has been shown to correlate with prognosis [26, 27]. Serum cholesterol level, reflecting a malnutritional and end stage liver function status, is a prognostic factor to predict postoperative HCC recurrence and OS in HCC patients as well [28].

Several limitations of the present study should be acknowledged. All the included studies were retrospective studies from Japan and China, using different cut-off values for the CONUT score, and with the different prevalence in patients with high CONUT score ranging from 9 to 49%. The number of included studies in the meta-analysis was small. Therefore, further studies are needed to identify the significance of the CONUT score and determine the most appropriate cut-off value to estimate the prognosis and complication risks in HCC patients.

## Conclusions

The present study suggests that the CONUT score could be an indicator to predict the prognosis, postoperative complications and hepatic functional reserve in patients following hepatectomy for HCC.

## Supplementary information

**Additional file 1: Table S1.** Search strings and terms**. Table S2.** The Newcastle-Ottawa scale for quality assessment of include studies. **Figure S1.** Funnel plots demonstrating primary endpoint in terms of low CONUT versus high CONUT score. (a) OS; and (b) RFS.

## Data Availability

The data supporting the conclusions of this article are included in this published article.

## Abbreviations

CDc: Clavien–Dindo classification
CONUT: Controlling nutritional status
HCC: Hepatocellular carcinoma
HR: Hazard ratio
ICGR15: Indocyanine green retention test after 15 min
MD: Mean difference
OR: Odds ratio; 95%CI: 95% confidence interval
OS: Overall survival
PHR: Postoperative hepatitis B reactivation
PNI: Prognostic nutritional index
RFS: Recurrence/relapse-free survival
