# Supplementary material for: Prognostic significance of the controlling nutritional status (CONUT) score in patients undergoing hepatectomy for hepatocellular carcinoma: a systematic review and meta-analysis
Source: BMC Gastroenterol. 2019 Dec 9;19:211. doi: 10.1186/s12876-019-1126-6 (PMC6902571; doi:10.1186/s12876-019-1126-6)

**Supplementary information**

**Table S1.** Search strings and terms

| **Database** | **Search query** | **Number of found records** | **Number of found records without duplication** |
| --- | --- | --- | --- |
| embase.com | ('controlling nutritional status score'/de OR 'controlling nutritional status'/de OR ('serum albumin'/de AND 'cholesterol blood level'/exp AND 'lymphocyte count'/exp) OR ('Controlling Nutritional Status' OR conut OR (albumin NEAR/10 cholesterol NEAR/10 lymphocyte NEAR/10 (count* OR serum* OR concentrat*))):ab,ti) AND ('digestive system tumor'/exp OR 'gastrointestinal surgery'/exp OR 'esophagus surgery'/exp OR (((digestive-system* OR gastr* OR intest* OR colo* OR hepat* OR pancrea* OR biliar* OR liver OR esophag* OR oesophag* OR rectal* OR rectum OR stomach OR hpb) NEAR/3 (tumor* OR tumour* OR cancer* OR malign* OR carcinom* OR adenocarcinom* OR surg* OR resect*)) OR hepatectom* OR pancreatectom* OR colectom* OR gastrectom* OR esophagectom* OR oesophagectom* OR pancreaticoduodenectom*):ab,ti) | 116 | 113 |
| Medline Ovid | ((Serum Albumin/ AND exp cholesterol/bl AND exp Lymphocyte Count/) OR (Controlling Nutritional Status OR conut OR (albumin ADJ10 cholesterol ADJ10 lymphocyte ADJ10 (count* OR serum* OR concentrat*))).ab,ti.) AND (exp Gastrointestinal Neoplasms/ OR exp Digestive System Surgical Procedures/ OR (((digestive-system* OR gastr* OR intest* OR colo* OR hepat* OR pancrea* OR biliar* OR liver OR esophag* OR oesophag* OR rectal* OR rectum OR stomach OR hpb) ADJ3 (tumor* OR tumour* OR cancer* OR malign* OR carcinom* OR adenocarcinom* OR surg* OR resect*)) OR hepatectom* OR pancreatectom* OR colectom* OR gastrectom* OR esophagectom* OR oesophagectom* OR pancreaticoduodenectom*).ab,ti.) | 69 | 8 |
| Web of science | TS=((("Controlling Nutritional Status" OR conut OR (albumin NEAR/10 cholesterol NEAR/10 lymphocyte NEAR/10 (count* OR serum* OR concentrat*)))) AND ((((digestive-system* OR gastr* OR intest* OR colo* OR hepat* OR pancrea* OR biliar* OR liver OR esophag* OR oesophag* OR rectal* OR rectum OR stomach OR hpb) NEAR/2 (tumor* OR tumour* OR cancer* OR malign* OR carcinom* OR adenocarcinom* OR surg* OR resect*)) OR hepatectom* OR pancreatectom* OR colectom* OR gastrectom* OR esophagectom* OR oesophagectom* OR pancreaticoduodenectom*))) | 63 | 13 |
| Cochrane CENTRAL | (('Controlling Nutritional Status' OR conut OR (albumin NEAR/10 cholesterol NEAR/10 lymphocyte NEAR/10 (count* OR serum* OR concentrat*))):ab,ti) AND ((((digestive-system* OR gastr* OR intest* OR colo* OR hepat* OR pancrea* OR biliar* OR liver OR esophag* OR oesophag* OR rectal* OR rectum OR stomach OR hpb) NEAR/3 (tumor* OR tumour* OR cancer* OR malign* OR carcinom* OR adenocarcinom* OR surg* OR resect*)) OR hepatectom* OR pancreatectom* OR colectom* OR gastrectom* OR esophagectom* OR oesophagectom* OR pancreaticoduodenectom*):ab,ti) | 18 | 11 |
| Google scholar | "Controlling Nutritional Status"\|conut "gastric\|gastrointestinal\|intestal\|colonic\|colorectal\|pancreatic\|liver\|esophageal tumor\|tumour\|cancer\|malignancy\|carcinoma\|resection\|hepatectomy\|pancreatectomy\|colectomy\|gastrectomy\|esophagectomy\|oesophagectomy" | 100 | 82 |
| Total |  | **366** | **227** |

**Table S2.** The Newcastle-Ottawa scale for quality assessment of include studies.

| **Study** | **Selection** | | | | **Comparability** | **Outcome** | | | **Total score** |
| --- | --- | --- | --- | --- | --- | --- | --- | --- | --- |
|  | Representativeness  of the exposed  cohort | Selection  of the  non-exposed  cohort | Ascertainment  of exposure | Demonstration  that outcome  of interest was  not present at  start of study | Comparability  of cohorts on  the basis of  the design or  analysis | Assessment  of outcome | Was follow-  up long  enough for  outcomes to occur | Adequacy  of follow  up of  cohorts |  |
| Total score | 1 | 1 | 1 | 1 | 2 | 1 | 1 | 1 | 9 |
| Takagi et al.[11] | 1 | 1 | 1 | 1 | 0 | 1 | 1 | 1 | 7 |
| Harimoto et al. [12] | 1 | 1 | 1 | 0 | 0 | 1 | 1 | 1 | 6 |
| Takagi et al. [13] | 1 | 1 | 1 | 0 | 1 | 1 | 1 | 0 | 6 |
| Harimoto et al. [14] | 1 | 1 | 1 | 0 | 0 | 1 | 1 | 1 | 6 |
| Li et al. [15] | 1 | 1 | 1 | 0 | 2 | 1 | 1 | 1 | 8 |
| Wang et al. [16] | 1 | 1 | 1 | 0 | 0 | 1 | 1 | 1 | 6 |
| Lin et al. [17] | 1 | 1 | 1 | 0 | 2 | 1 | 1 | 1 | 8 |

**Figure S1.** Funnel plots demonstrating primary endpoint in terms of low CONUT versus high CONUT score. (a) OS; and (b) RFS.

1. OS


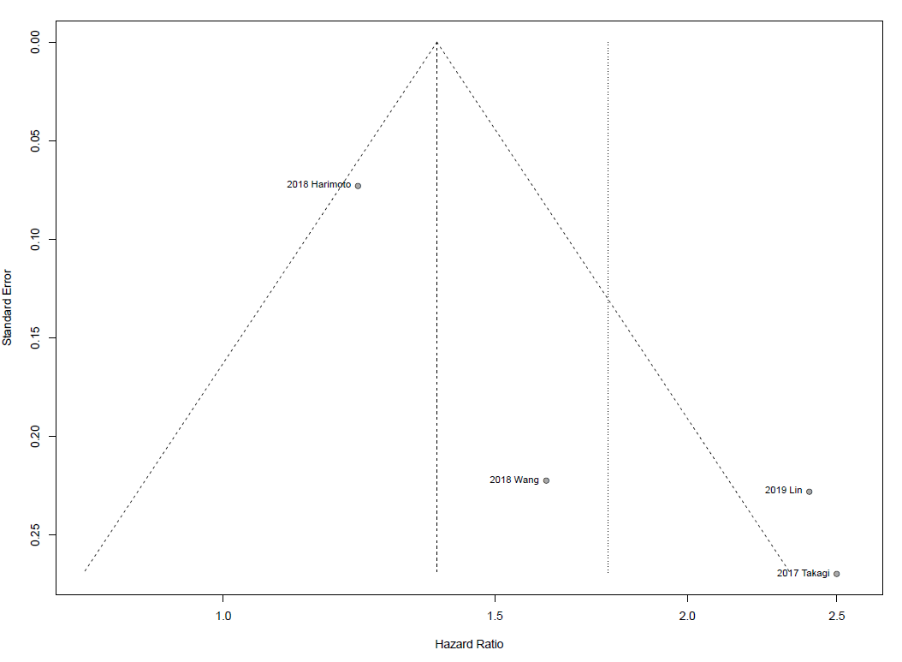


1. RFS


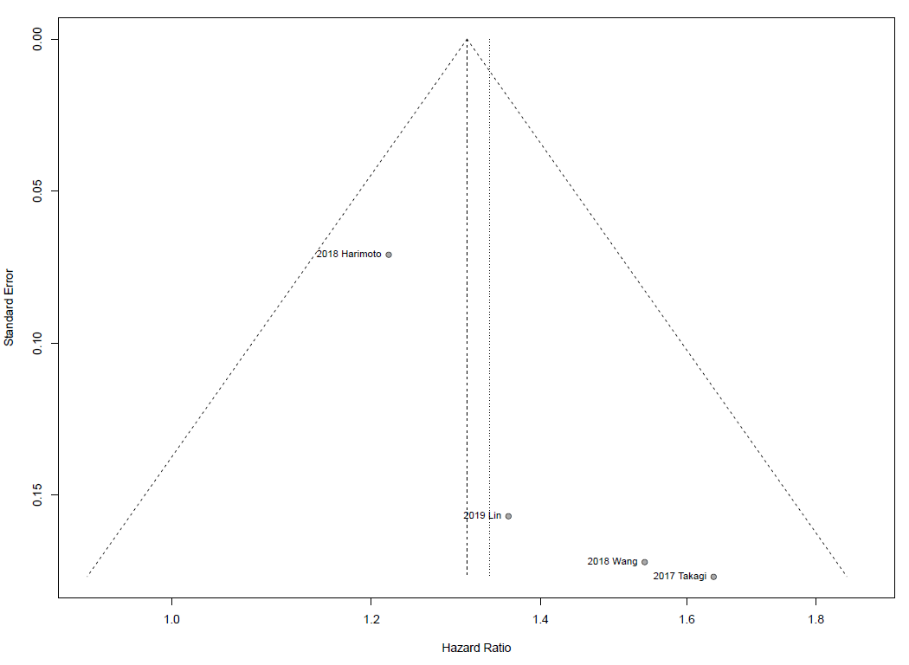

Supplement: Supplementary file 1 — Additional file 1: Table S1. Search strings and terms. Table S2. The Newcastle-Ottawa scale for quality assessment of include studies. Figure S1. Funnel plots demonstrating primary endpoint in terms of low CONUT versus high CONUT score. (a) OS; and (b) RFS. [file 12876_2019_1126_MOESM1_ESM.docx]
